# High Glucose Induces the Loss of Retinal Pericytes Partly via NLRP3-Caspase-1-GSDMD-Mediated Pyroptosis

**DOI:** 10.1155/2020/4510628

**Published:** 2020-04-23

**Authors:** Jinhua Gan, Maomao Huang, Genyin Lan, Li Liu, Fangyuan Xu

**Affiliations:** ^1^Department of Ophthalmology, Affiliated Hospital of Southwest Medical University, Luzhou, Sichuan, China 646000; ^2^Department of Rehabilitation, Affiliated Hospital of Southwest Medical University, Luzhou, Sichuan, China 646000

## Abstract

Diabetic retinopathy (DR) is one of the hallmark complications of diabetes and a leading cause of vision loss in adults. Retinal pericyte death seems to be a prominent feature in the onset of DR. Pyroptosis is an inflammatory form of programmed cell death, defined as being caspase-gasdermin-D (GSDMD)-dependent. The NOD-like receptor pyrin 3 (NLRP3) inflammasome plays an important role in mediating GSDMD activation. However, the role and mechanism of pyroptosis in the loss of retinal pericytes during the pathogenesis of DR are still unclear. In the present study, we cultured primary human retinal pericytes (HRPs) in high glucose medium; caspase-3 inhibitor DEVD, caspase-1 inhibitor YVAD, or NLRP3 inhibitor glyburide was used as intervention reagents; GSDMD was overexpressed or suppressed by transfection with an expressing vector or retroviral silencing of GSDMD, respectively. Our data showed that high glucose induced NLRP3-caspase-1-GSDMD activation and pore formation in a dose- and time-dependent manner (*p* < 0.05) and resulted in the inflammatory cytokines IL-1*β* and IL-18 and lactate dehydrogenase (LDH) release from HRPs (*p* < 0.05), which are all signs of HRP pyroptosis. Overexpression of GSDMD facilitated high glucose-induced pyroptosis (all *p* < 0.05). However, these effects were blunted by synergistically treating DEVD, YVAD, and silencing GSDMD (*p* < 0.05). Taken together, our results firstly revealed that high glucose induced the loss of retinal pericytes partly via NLRP3-caspase-1-GSDMD-mediated pyroptosis.

## 1. Introduction

DR is a frequent retinal microvascular complication and a leading cause of vision loss in adults [1]. The loss of retinal pericytes is one of the earliest changes associated with DR, and it has been postulated to initiate or trigger microaneurysm formation, abnormal leakage, edema, and ischemia, provoking proliferative neovascularization in the retina [2, 3]. Although it has been observed in diabetic patients and in animal models of DR, the cause of pericyte death remains unknown [4, 5].

Previous studies found that exposure of pericytes to high glucose reduced their proliferation and induced apoptosis [6]. Recently, an emerging type of cell death “pyroptosis” has caught everyone's attention. Pyroptosis is a unique, proinflammatory form of lytic cell death that is initiated by the activation of proinflammatory caspases (caspase-1, caspase-4, caspase-5, and caspase-11) [7]. The NLRP3 inflammasome, the best characterized inflammasome to date, contains the NLRP3 associated with the adapter protein, apoptosis-associated speck-like protein, and caspase-1 [8]. Proinflammatory caspases cleave a GSDMD protein to generate a 31 kDa gasdermin-N domain (GSDMD-N) via the canonical and noncanonical inflammasome signaling pathways, which then moves to the plasma membrane to exhibit pore-forming activity [9, 10]. Thus, GSDMD-N acts as the final and direct executor of pyroptosis. Recent research reported that inhibition of the caspase-1/IL-1*β* pathway prevented diabetes-induced Müller cell loss, suggesting that pyroptosis could be involved in the process of Müller cell death [11]. However, the role of pyroptosis in the loss of retinal pericytes during the pathogenesis of DR is still unclear.

In the present study, we cultured primary HRPs in high glucose medium; caspase-3 inhibitor DEVD was used to inhibit caspase-3-mediated apoptosis, caspase-1 inhibitor YVAD or NLRP3 inhibitor glyburide was used to block NLRP3-caspase-1 signaling, and GSDMD-N was overexpressed or suppressed by adenoviral vectors or small hairpin RNA (shRNA); we observed whether high glucose induced the pore formation and pyroptosis via NLRP3-caspase-1-GSDMD signaling, aiming to elucidate the role and molecular mechanism of pyroptosis in the pathogenesis of DR.

## 2. Materials and Methods

### 2.1. Cell Culture and Treatment

The primary HRPs (ACBRI 183) were obtained from Cell Systems Corporation (Kirkland, WA, USA) and maintained at 37°C in a humidified atmosphere containing 5% CO_2_ in low-glucose Dulbecco's Modified Eagle Medium (DMEM) with 10% serum (Gibco, CA, USA) and Attachment Factor (CS-4Z0-500; Cell Systems, Kirkland, WA, USA), supplemented with 100 U/mL penicillin and 100 *μ*g/mL streptomycin (Beyotime, China).

The HRPs were used for all of the experiments and randomly divided into the following treatments: (1) normal control group (NC group; with medium that contained 5.6 mmol/L glucose); (2) high glucose group (HG group; 10, 20, or 30 mmol/L (mM) glucose); and (3) osmotic pressure group (OP group; with medium that contained 5.6 mmol/L glucose+24.6 mmol/L mannitol as control). Before high glucose stimulation, 1, 25, 50, and 100 *μ*mol/L (*μ*M) caspase-3 inhibitor Z-DEVD-FMK (DEVD, Sigma, No. 264156), 1, 5, 10, and 20 *μ*M caspase-1 inhibitor Z-YVAD-FMK (YVAD, Santa Cruz, sc-3071), or 200 *μ*M NLRP3 inhibitor glibenclamide (glyburide, Santa Cruz, sc-200982) was added into the culture medium [12]. After cells were induced for 12, 24, or 48 h, the culture supernatant of each group was collected, and the total proteins were extracted for further study.

### 2.2. Cell Viability Assay

Initially, to determine the proper concentrations of these regents, the viability of the cells was determined by quantitative colorimetric assay with 3-(4,5-dimethylthiazol-2-yl)-2,5-diphenyltetrazolium bromide (MTT) in 96-well plates. Absorbance at 570 nm was measured for the experimental groups using a microplate reader. According to the MTT results, 30 mM high glucose, 50 *μ*M DEVD, and 10 *μ*M YVAD were used in the entire study.

### 2.3. Pore Formation Assay

Pore formation was quantified based on the permeability of propidium iodide (PI) in damaged cells as previously described [13]. HRPs were seeded in a black, clear-bottom 96-well plate (1 × 10^5^ cells/well). Before incubation with different compounds as described above, the medium was replaced with DMEM complete medium without phenol red with 20 mM HEPES and 6 *μ*L/mL PI. After incubation for 48 h, HRPs were maintained at 37°C, and PI was excited at 538 nm. The fluorescence emission was read at 617 nm every 6 hours using a plate fluorometer (SpectraMax i3x, Molecular Devices). Pore formation was assessed fluorometrically in real time by the uptake of PI; relative fluorescence units (RFU %) represents the percentage of RFU estimated with cells lysed with 0.1% Triton X-100, and the incubation time of Triton X-100 was the same as that of the experimental group.

### 2.4. Cell Death Assays

At the indicated times, supernatants were harvested for analysis of LDH released by dying cells. LDH levels in the supernatants were quantified using the CytoTox 96 LDH-release kit (Promega). The LDH release (%) represents the percentage of LDH release estimated with cells lysed with 0.1% Triton X-100 at the same incubation time.

### 2.5. Exogenous GSDMD-N Overexpression Vector Construction and *In Vitro* Cell Treatment

Open reading frames of human GSDMD-N were amplified using cDNA from HeLa cells by PCR and cloned to the C terminus of GFP-pEGFP-C1 (Takara Biomedical Technology) to construct the GFP-GSDMD-N expression vector by standard cloning procedures. pEGFP-C1 is a control plasmid that express GFP but cannot overexpress GSDMD-N protein. Adenoviral vectors were generated and purified, and all inserted genes were sequenced. HRPs were infected with the GFP-NLRP3 expression vector or pEGFP-C1 control vector and then were stimulated by 30 mM high glucose for 48 h. GFP fluorescence images were taken with a laser scanning confocal microscope (Leica, Germany), and the values of semiquantitative analysis for average intensity of GFP were assessed by Image-Pro Plus 6.0 software.

### 2.6. Retroviral Silencing of GSDMD

HRPs were transfected with lentiviral vectors encoding shRNA targeting GSDMD Seq1: (Sequence-CCGGGATTGATGAGGAGGAATTAATCTCGAGATTAATTCCTCCTCATCAATCTTTTTG, Sigma-TRCN0000219619), Seq2: (Sequence-CCGGCCTAAGGCTGCAGGTAGAATCCTCGAGGATTCTACCTGCAGCCTTAGGTTTTTG, Sigma-TRCN0000219620), and a nontarget shRNA sequence. Lentiviruses expressing shRNAs were collected and infect HRPs at a multiplicity of infection of 10. The GSDMD silencing efficiency was confirmed by western blotting after 12 hours, and HRPs were then stimulated by 30 mM high glucose alone or were intervened by 50 *μ*M DEVD or 10 *μ*M YVAD for 48 h; the cells and culture medium were collected for ELISA and western blotting analysis.

### 2.7. Enzyme-Linked Immunosorbent Assay (ELISA)

IL-1*β* and IL-18 protein levels in the culture supernatant were determined using commercially available ELISA IL-1*β* and IL-18 kits (NeoBioscience, China) according to the manufacturer's protocols. The results were determined by comparing the samples to the standard curve generated by the kit.

### 2.8. Protein Extraction and Western Blotting

Total proteins were isolated from HRPs using a BCA Protein Assay Kit (Beyotime, China). Proteins were separated by sodium dodecyl sulfate-polyacrylamide gel electrophoresis and transferred to a polyvinylidene difluoride membrane (Millipore). Immunoblotting was performed using NLRP3 antibody (mouse monoclonal antibody; dilution 1 : 1000; Abcam, No. Ab16097), caspase-1 antibody (rabbit monoclonal antibody; dilution 1 : 800; Cell Signaling Technology, No. 3866), caspase-4 antibody (rabbit monoclonal antibody; dilution 1 : 600; Cell Signaling Technology, No. 4450), caspase-5 p20 antibody (mouse monoclonal antibody; dilution 1 : 600; Santa Cruz, sc-393346), cleaved IL-1*β* antibody (rabbit monoclonal antibody; dilution 1 : 1000; Cell Signaling Technology, No. 83186), GSDMD (rabbit polyclonal antibody; dilution 1 : 1000; Cell Signaling Technology, No. 96458), and anti-*β*-actin antibody (mouse monoclonal antibody; dilution 1 : 2000; Beyotime, China).

### 2.9. Data Analysis

All data obtained from at least three independent experiments were expressed as the mean ± standard deviation (SD) and analyzed using two-tailed indirect Student's *t*-test or one-way analysis of variance (ANOVA), followed by the LSD post hoc test for multiple comparisons (SPSS 18.0 statistical software). *p* < 0.05 was considered significant.

## 3. Result

### 3.1. High Glucose Induced Inflammation, Pore Formation, and Cell Death

**Figure 1:**
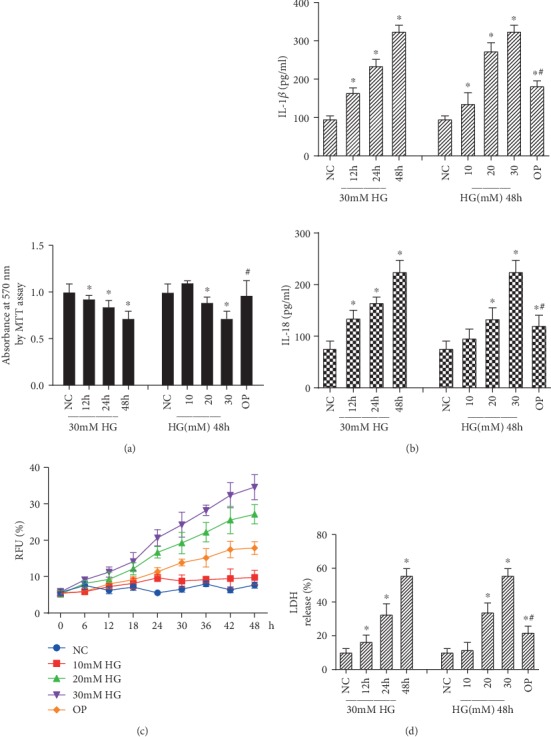
High glucose induced inflammation, pore formation, and cell death. HRPs were firstly treated with 30 mM glucose for 12, 24, and 48 h or were treated with the indicated concentrations of high glucose (HG) or mannitol (osmotic pressure control, OP) for 48 h. (a) Cell viability was determined by MTT analysis. (b) IL-1*β* and IL-18 levels in the cell culture supernatant were determined by ELISA. (c) Fluorometric plots show PI uptake (RFU (%)) over time to reveal the kinetics of pore formation. (d) Cell death was monitored by measuring LDH released (LDH release (%)) by dying cells. Data are expressed as mean ± SD of triplicate wells or one representative experiment of three similar results. ^∗^*p* < 0.05 compared with the NC group. ^#^*p* < 0.05 compared with the 30 mM HG group.

The MTT results showed that 30 mM high glucose could inhibit the proliferation of HRPs compared with NC and OP groups in a time- and dose-dependent manner (*p* < 0.05) (Figure 1(a)). Meanwhile, compared with the NC group and the OP group, the inflammatory cytokine IL-1*β* and IL-18 releases from HRPs were obviously induced by high glucose (*p* < 0.05) (Figure 1(b)), indicating that high glucose would lead to severe inflammatory state and cell death. It should be pointed out that a significant difference was also found between the OP and NC groups; however, IL-1*β* and IL-18 levels were significantly decreased in the OP group compared with the 20 mM and 30 mM glucose groups (*p* < 0.05), suggesting that osmotic pressure had little effect on the high glucose-induced inflammatory state. Next, RFU (%) was monitored in real time to assess membrane permeabilization fluorometrically via the uptake of PI; the results showed that HRPs triggered robust pore formation in response to high glucose in a dose-dependent manner (*p* < 0.05) (Figure 1(c)). High glucose-induced pore formation culminates with the induction of HRP lysis. Thus, we measured LDH release in the supernatants of HRPs and found that high glucose-induced HRP lysis occurred in a time- and dose-dependent manner (Figure 1(d)). Collectively, these data support the hypothesis that compared with hyperosmotic state, high glucose itself is more likely to induce inflammation, pore formation, and cell death.

### 3.2. High Glucose Induced Inflammation and Pyroptosis in Part via NLRP3-Caspase-1-GSDMD Signaling

**Figure 2:**
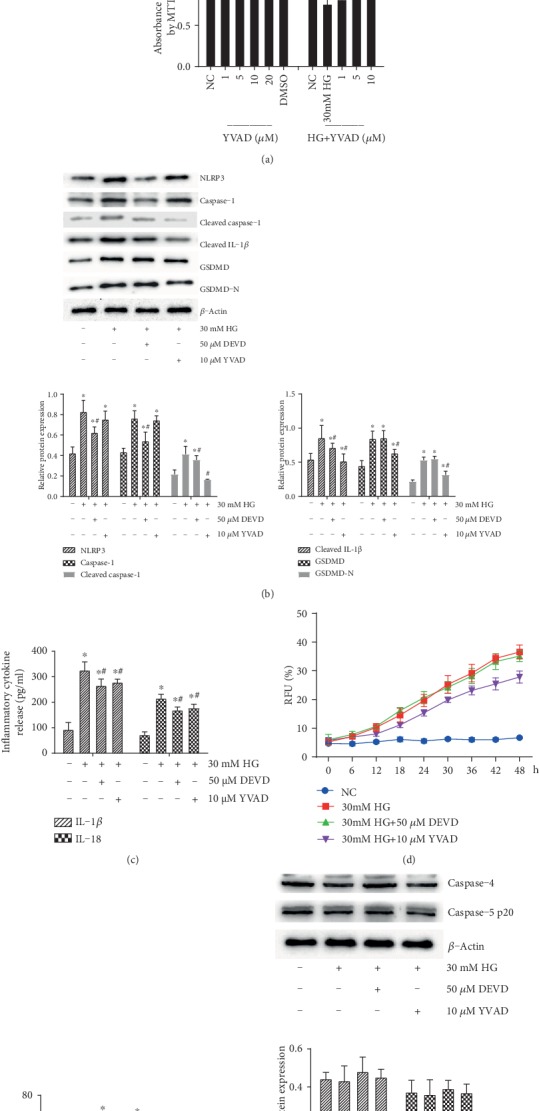
High glucose induced inflammation and pyroptosis in part via NLRP3-caspase-1-GSDMD signaling. HRPs were firstly incubated with 30 mM high glucose (HG) alone or were treated with the indicated concentrations of caspase-3 inhibitor DEVD or caspase-1 inhibitor YVAD following 30 mM HG for 48 h. (a) Cell viability was determined by MTT analysis. (b) The expression of NLRP3, caspase-1, cleaved caspase-1, cleaved IL-1*β*, GSDMD, and GSDMD-N in lysates of HRPs was detected by western blotting. (c) IL-1*β* and IL-18 levels in the cell culture supernatant were determined by ELISA. (d) Fluorometric plots show PI uptake (RFU (%)) over time to reveal the kinetics of pore formation. (e) LDH release (LDH release (%)) was measured by LDH-release kit. (f) The expression of caspase-4 and caspase-5 p20 in lysates of HRPs was detected by western blotting. Data are expressed as mean ± SD. ^∗^*p* < 0.05 compared with the NC group. ^#^*p* < 0.05 compared with the 30 mM HG group.

In order to evaluate which signal is involved in high glucose-induced inflammation, pore formation, and pyroptosis, the effect of different concentrations of caspase-3 inhibitor DEVD or caspase-1 inhibitor YVAD on HRP viability was also assessed, respectively, by MTT. We observed that both the 1-50 *μ*M concentration range of DEVD and the 1-10 *μ*M concentration range of YVAD reversed high glucose-inhibited proliferation in a dose-dependent manner (*p* < 0.05) (Figure 2(a)). Furthermore, 50 *μ*M DEVD or 10 *μ*M YVAD significantly reversed high glucose-induced NLRP3, caspase-1, cleaved caspase-1, and cleaved IL-1*β* expression (Figure 2(b)) and the abnormity of cytokines IL-1*β* and IL-18 (Figure 2(c)) release from HRPs (*p* < 0.05). However, high glucose-induced activation of GSDMD was only reversed by YVAD (*p* < 0.05), but not DEVD (*p* > 0.05). Consistent with these findings, high glucose-mediated pore formation (Figure 2(d)) and LDH release (Figure 2(e)) were abrogated by YVAD (*p* < 0.05), but not DEVD (*p* > 0.05). Next, the results showed that the key proteins of noncanonical pyroptosis pathway, caspase-4 and caspase-5 p20 expression, were not changed by high glucose, DEVD, or YVAD (*p* > 0.05) (Figure 2(f)), suggesting that high glucose induced inflammation and cell pyroptosis in part via caspase-1, but not caspase-4 or caspase-5.

### 3.3. Overexpression of GSDMD-N Facilitated High Glucose-Induced Inflammation and Pyroptosis

**Figure 3:**
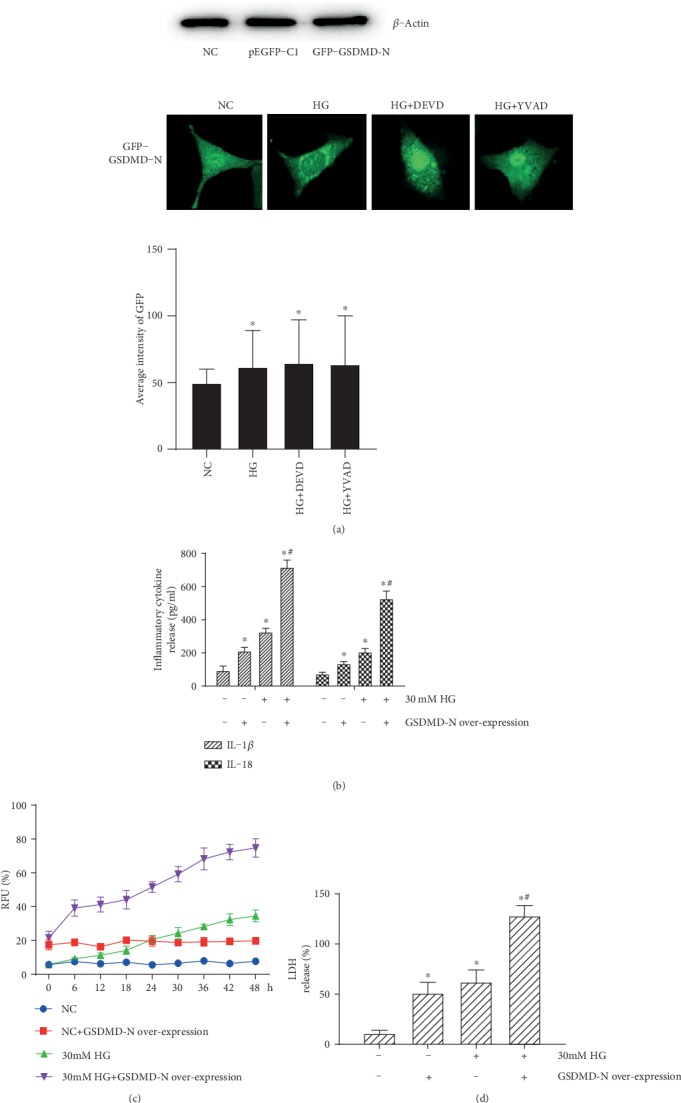
Overexpression of GSDMD-N facilitated high glucose-induced inflammation and pyroptosis. Plasmids expressing GSDMD-N with an N-terminal GFP tag, and pEGFP-C1 control plasmid that express GFP but cannot overexpress GSDMD-N, were constructed to determine the effect of overexpressed GFP-GSDMD-N fusion protein in HRPs under 30 mM high glucose (HG) stimulation and DEVD or YVAD treatment by western blotting or fluorescence images (×400). High glucose-induced (b) IL-1*β* and IL-18 release from *HRPs*, (c) pore formation, and (d) LDH release were significantly facilitated by the overexpression of GSDMD-N. Data are expressed as mean ± SD. ^∗^*p* < 0.05 compared with the NC group. ^#^*p* < 0.05 compared with the 30 mM HG group.

Plasmids overexpressing human GSDMD-N with a GFP tag or pEGFP-C1 control plasmid were constructed to determine the effects of overexpressed GSDMD-N on high glucose-mediated inflammation and HRP pyroptosis. The results showed that the GFP-GSDMD-N fusion protein was overexpressed, especially in HRPs of 30 mM high glucose group (*p* < 0.05), and its expression was not reversed by DEVD or YVAD; interestingly, we found that fluorescence of GFP-GSDMD-N fusion protein concentrates into the cell nucleus after DEVD or YVAD treatment, but the reason was unknown (Figure 3(a)). To assess the involvement of GSDMD-N in the regulation of high glucose-induced inflammation, pore formation, and cell death, IL-1*β* and IL-18 secretion (Figure 3(b)), RFU (%) (Figure 3(c)), and LDH release (%) (Figure 3(d)) were detected; the results showed that consistent with the GFP-GSDMD-N fusion protein expression, 30 mM high glucose-mediated changes were significantly facilitated by overexpressed GFP-GSDMD-N (*p* < 0.05). Taken together, these data suggested that overexpression of GSDMD-N facilitated the high glucose-induced inflammation and HRP pyroptosis.

### 3.4. shRNA-GSDMD in Part Reversed the High Glucose-Induced Inflammation and Pyroptosis

**Figure 4:**
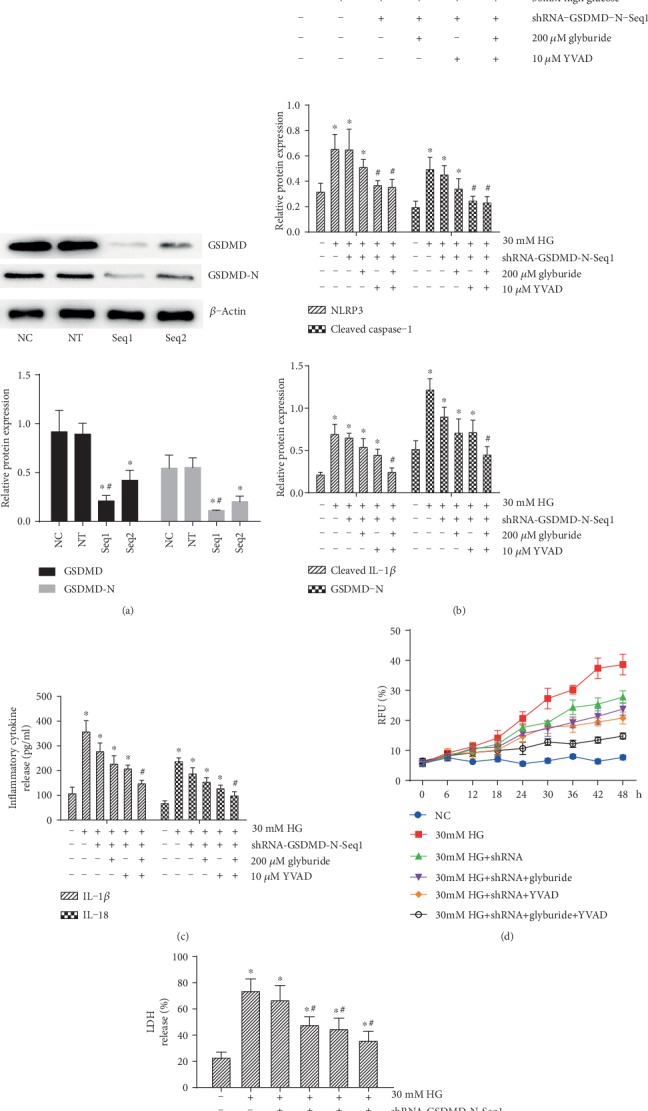
shRNA-GSDMD in part reversed the high glucose-induced inflammation and pyroptosis. (a) Western blotting detected the protein expression of GSDMD and GSDMD-N in HRPs treated with shRNA-GSDM seq1 or shRNA-GSDM seq2. 200 *μ*M glyburide or 10 *μ*M YVAD was used as intervention reagents; 30 mM high glucose- (HG-) induced (b) NLRP3, cleaved caspase-1, cleaved IL-1*β*, and GSDMD-N expression, (c) IL-1*β* and IL-18 release, (d) pore formation, and (e) LDH release were detected by western blotting, ELISA, PI staining, or LDH-release kit, respectively. Data are expressed as mean ± SD. ^∗^*p* < 0.05 compared with the NC group. ^#^*p* < 0.05 compared with the 30 mM HG group.

We established the shRNA-GSDMD to realize silencing of GSDMD of HRPs. As shown in Figure 4(a), the protein expression levels of GSDMD and GSDMD-N were significantly inhibited by shRNA-seq1 and shRNA-seq2 compared with the nontarget shRNA sequence (NT) group and NC group (*p* < 0.05). Considering the relative expression of GSDMD, we chose shRNA-seq1 in the following studies. Next, the 200 *μ*M NLRP3 inhibitor glyburide or 10 *μ*M caspase-1 inhibitor YVAD was used to inhibit NLRP3-caspase-1 signaling; the expression of NLRP3, cleaved caspase-1, cleaved IL-1*β*, and GSDMD-N was detected by western blotting; the results showed that 30 mM high glucose-mediated NLRP3-caspase-1 activation was not blunted by shRNA-GSDMD unless combined with glyburide or YVAD (Figure 4(b)). Similarly, although shRNA-GSDMD has proven effective in inhibiting the IL-1*β* and IL-18 release (Figure 4(c)), RFU (%) (Figure 4(d)) and LDH release (%) (Figure 4(e)) revealed that the silencing of GSDMD-inhibited pyroptosis was significantly facilitated by synergistically treating glyburide or YVAD, suggesting that high glucose induced the loss of HRPs via NLRP3-caspase-1-GSDMD-mediated pyroptosis.

## 4. Discussion

Diabetes-induced cell death has been observed in numerous retinal cell types such as endothelial cells [14, 15], pericytes [16, 17], neural retinal cells such as ganglion cells [18, 19], and retinal glial cells such as Müller cells, astrocytes, and microglia [20, 21]. Pyroptosis is an inflammatory form of programmed cell death distinguished from apoptosis, ferroptosis, necrosis, necroptosis, netosis, oncosis, pyronecrosis, and autophagy [6, 7]. Execution of pyroptosis might depend on cell type, microenvironment, and stimulus. Previous studies clearly demonstrated that glial cells might respond to chronically elevated glucose levels by pyroptosis [11, 22]. More studies are needed to fully understand the mechanism of pyroptosis and to determine whether HRPs are undergoing pyroptosis in diabetes. Here, our data showed that compared with hyperosmotic state, high glucose itself induced HRP inflammation, pore formation, and pyroptosis in a time- and dose-dependent manner. These results suggested that, in addition to apoptosis, pyroptosis might play an important role in the loss of retinal pericytes in the pathogenesis of DR. Therefore, it is of great significance to inhibit the pyroptosis of retinal pericytes for the treatment of early stages of DR.

During pyroptosis, there is an assembly of a multiprotein platform allowing for caspase-1 activation that is termed either the inflammasome or pyroptosome [23]. The canonical NLRP3 inflammasome activation leads to autoprocess caspase-1 then mature the proinflammatory cytokines IL-1*β* and IL-18 to render them bioactive and induce pyroptosis [24]. Besides this canonical pathway, other proinflammatory caspases, murine caspase-11 and human caspase-4 and caspase-5, directly sense cytosolic LPS to induce pyroptosis and activate the NLRP3 inflammasome via the so-called noncanonical pathway [25, 26]. Our results showed that high glucose induced NLRP3-caspase-1 inflammasome signaling, but not caspase-4 or caspase-5, and NLRP3 inhibitor glyburide or caspase-1 inhibitor YVAD ameliorated high glucose-induced pyroptosis, suggesting that high glucose induced HRP pyroptosis in part via the canonical NLRP3-caspase-1 pathway.

Under steady-state conditions, full-length GSDMD was exclusively localized in the cytosol, inflammatory caspases cleaved GSDMD to generate GSDMD-N, which is capable of forming porelike structures in lipid membranes and thus constitutes the direct and sole effector of pyroptosis [27, 28]. Furthermore, GSDMD-N alone induced pyroptosis when expressed ectopically [29]. So far, all studies come to the conclusion that the pore-forming property of the GSDMD-N is the driver of pyroptosis. Our results revealed that both GSDMD and GSDMD-N expression was increased by high glucose, but NLRP3 inhibitor glyburide, or caspase-1 inhibitor YVAD, reversed high glucose-induced GSDMD activation, pore formation, and pyroptosis; overexpression of GSDMD-N facilitated the high glucose-induced pyroptosis, suggesting that GSDMD, as the sole pyroptosis effector molecule, is capable of building pores in lipid membranes in response to high glucose-induced NLRP3-caspase-1 pathway activation.

It must be pointed out that high glucose-mediated NLRP3-caspase-1 activation was not blunted by shRNA-GSDMD alone unless combined with glyburide or YVAD, and shRNA-GSDMD-inhibited anti-inflammation and antipyroptosis effects were facilitated significantly by treating glyburide or YVAD, suggesting that there must be cell death mechanisms, such as apoptosis, involved in the cross talk of NLRP3-caspase-1 signaling and inflammatory form of programmed cell death during high glucose stimulation [11, 30]. Therefore, our studies by no means rule out other potential mechanisms by which high glucose may induce the loss of retinal pericytes, such as apoptosis, necroptosis, or autophagic cell death. Previous studies also found that high glucose-induced HRP apoptosis depends on the association of inflammatory signaling and GAPDH, and *Homonoia riparia* and its major component, myricitrin, inhibit high glucose-induced apoptosis of HRPs [6, 31]. In addition, this study did not explain the reason of GFP-GSDMD-N fusion protein concentration into the cell nucleus after DEVD or YVAD treatment; we speculated that the reason may be that DEVD or YVAD affected the cell cycle; it may be an interesting subject for future investigation involved in DEVD or YVAD. Even so, our study suggests a new type of the loss of retinal pericytes in response to high glucose stimulation via NLRP3-caspase-1-GSDMD-mediated pyroptosis. In view of the fact that the regulatory mechanisms of NLRP3-caspase-1-GSDMD signaling are extremely complex, future studies will focus on the pyroptosis-related specific mechanisms; studies *in vivo* will be necessary to evaluate the therapeutic potential of targeting the NLRP3-caspase-1-GSDMD pathway in different stages of DR and to understand adverse side effects associated with inhibiting retinal pericyte pyroptosis.

In conclusion, the present study found that high glucose induced the loss of retinal pericytes in part via NLRP3-caspase-1-GSDMD-mediated pyroptosis. These results help to clarify the association between pyroptosis and high glucose stimulation, suggesting a new target for DR and diabetic complications.

## Data Availability

This manuscript is not under consideration for publication elsewhere. Publication of the article is approved by all authors and tacitly or explicitly approved by the responsible authorities where the work was performed. If the manuscript is accepted, it will not be published elsewhere by the authors, including electronically in the same form, in English or in any other language, without the written consent of the copyright holder.
